# Trends in mortality due to diabetes in Brazil, 1996–2011

**DOI:** 10.1186/s13098-015-0105-5

**Published:** 2015-11-26

**Authors:** Maria Inês Schmidt, Bruce B. Duncan, Lenice Ishitani, Glaura da Conceição Franco, Daisy Maria Xavier de Abreu, Gustavo C. Lana, Elisabeth França

**Affiliations:** Postgraduate Program in Epidemiology, School of Medicine, Federal University of Rio Grande do Sul, Rua Ramiro Barcelos, 2600/414, Porto Alegre, RS 90035-003 Brazil; Secretaria Municipal de Saúde de Belo Horizonte, Belo Horizonte, MG Brazil; Statistics Department, Federal University of Minas Gerais, Belo Horizonte, MG Brazil; Grupo de Pesquisa em Epidemiologia e Avaliação em Saúde-(GPEAS), Federal University of Minas Gerais, Belo Horizonte, MG Brazil; Postgraduate Program in Public Health, School of Medicine, Federal University of Minas Gerais, Belo Horizonte, MG Brazil

## Abstract

**Background:**

Over recent decades, Brazilian mortality registration has undergone increasing improvement in terms of completeness and quality in cause of death reporting. These improvements, however, complicate the description of mortality trends over this period. We aim to characterize the trend in diabetes mortality in Brazil and its five regions in adults (30–69 years), from 1996 to 2011 after corrections for underreporting of deaths and redistribution of ill-defined causes and “garbage codes”.

**Methods:**

Starting with official data from the Brazilian Mortality Information System (SIM) for adults aged 30–69 in the period 1996 to 2011 for diabetes (ICD-10 codes E10-14), we redistributed garbage codes using methods based on the Global Burden of Disease Study (2010), redistributed ill-defined causes based on recent Brazilian investigations of similar cases and corrected for underreporting using official estimates of deaths.

**Results:**

With these corrections, age-standardized mortality fell approximately 1.1 %/year for men and 2.2 %/year for women from 1996 to 2011. The rate of decline first accelerated and then decelerated, reaching stable rates in men and minimal declines in women from 2005 onward. Regional inequalities decreased during the period in both relative and absolute terms.

**Conclusion:**

Mortality due to diabetes declined in Brazil from 1996 to 2011, minimally in men and considerably in women. The lesser declines in recent years may reflect the increasing prevalence of diabetes, and suggest that current efforts to prevent diabetes and minimize the impact of its complications need to be reinforced to ensure that declines will continue.

## Background

Diabetes, a state of hyperglycemia defined by greater risk of microvascular damage (retinopathy, nephropathy and neuropathy), is increasingly recognized as an important public health problem. Diabetes reduces life expectancy, augments morbidity due to microvascular and macrovascular complications (ischemic heart disease, stroke and peripheral vascular disease), increases premature mortality, and diminishes the quality of life [1].

It has been estimated that 387 million people had diabetes in 2013 and that 592 million will have the disease in 2035. Approximately half of those with diabetes are under 60 years of age, and 77 % of those with diabetes live in low- and middle-income countries. This scenario is of great concern given that Type 2 diabetes, the most common form of the disease, is likely to continue to rise as a consequence of population ageing and urbanization, as well as of the current obesity epidemic, resulting in very high direct and indirect costs to individuals and to society [2]. Although not fully understood, the epidemic of diabetes may result not only from increased incidence, but also from improved survival. Improved survival has been demonstrated in some developing countries [3].

The Global Burden of Disease (GBD) Study reported a 9.0 % increase in standardized mortality from diabetes between 1990 and 2013, with diabetes progressing from the 26th to the 17th leading cause of years of life lost globally. In 2013 diabetes was the 7th cause of years of life lost in Brazil [4].

Scant data exist with respect to trends in diabetes mortality for Brazil, a high-middle income country in which known diabetes prevalence is 6.2 % [5], and in which approximately 50 % of diabetes is estimated to be undiagnosed [6]. Increases in diabetes mortality have been reported in recent decades [7–9], possibly due in part to a greater diabetes prevalence and better recognition of diabetes as a cause of death. These analyses, however, have not incorporated corrections for problems in mortality reporting.

Analyses of trends in mortality in Brazil remain a challenge. Although the Brazilian Mortality Information System (SIM) is universal and consolidated, coverage of deaths and quality of information on causes of death are unequal across space and time, with sub-enumeration of deaths and a high proportion of ill-defined causes among registered deaths in some areas [10, 11]. Thus, analyses of trends require the accounting of these factors to avoid bias in comparisons across regions and over time.

Analyses incorporating these corrections have found a sharp decrease in the age-standardized mortality for non-communicable diseases in recent decades [12], mainly due to falls in cardiovascular and chronic respiratory diseases. This could result in improved survival among diabetic individuals, and therefore contribute to the increased diabetes prevalence in Brazil. However, over the same period, declines in diabetes mortality were modest [13].

This study aims to characterize further the trend in diabetes mortality in Brazil and its five regions in adults (30–69 years), from 1996 to 2011, using the method for correction of under-registration of deaths currently recommended by the Ministry of Health together with a new method of reallocating ill-defined causes, redistributing not just those formally declared as ill-defined but also those initially reported within the so-called “garbage codes”.

## Methods

We used the Brazilian Mortality Information System (SIM) for the period 1996 to 2011 to obtain the reported numbers of deaths of adults aged 30–69 years from the public website of the Ministry of Health [14]. Procedures used were similar to those employed by the GBD2010 [15], unless indicated. Deaths whose underlying cause was diabetes were selected using all ICD-10 diabetes codes (E10.0–E14.9). After reallocating the small fraction of deaths with missing information for sex and age of death, data were corrected following three steps.

First, we considered as “garbage codes” all deaths from nonspecific causes within ICD-10 chapters of defined causes (i.e., all chapters except Chapter XVIII). We defined the fraction of each specific ICD-10 garbage code to be redistributed to “target” diabetes codes, separately by sex, age and region, adapted from the list of garbage codes of GBD-2010, adding deaths redistributed from these garbage codes to those directly reported as due to diabetes [15, 16].

Second, we redistributed codes from ill-defined causes of deaths (Chapter XVIII of ICD-10). We did this separately by sex, five-year age group and region, in the proportions similar to those found for diabetes during routine investigations carried out by state and local health departments in the country since 2006 [17].These proportions, here called IDC redistribution coefficients (RD-IDC), were defined for each year, region and sex. We used data from the same year’s investigation for redistribution in the years between 2006 and 2011, and the mean RD-IDCs over 2006–2011 for the period 1996–2005, which preceded this investigation of ill-defined causes.

Third, we corrected the numbers produced in the second step for underreporting of deaths for the years 1996 to 2011, by applying the inverse of the ratio of reported/estimated deaths by the Ministry of Health [18]. This step produced the corrected number of total deaths in each sex and five-year age group, in each geographical region.

We next produced mortality rates by applying population denominators obtained from the 1991, 2000 and 2010 Brazilian censuses from IBGE to these numbers of deaths. Intercensus population estimates by age and sex were obtained by logarithmic interpolation of the census population. We then performed direct standardization to the 2010 Brazilian population to produce yearly age standardized death rates (/100,000 population) overall and by region and sex.

We investigated time trends in these standardized mortality rates, from 1996 to 2011, with a linear regression model which assumes a constant (linear) trend over the series, to test the hypothesis of a positive or a negative trend (slope different from zero). To adjust for the presence of first order autocorrelation, the residuals of the regression were modeled as a first order autoregressive process [19, 20]. It was then possible to test if the mortality series presents a significant increasing or decreasing trend. Finally, to explore nuances in trends, we used a state space model [21], which does not assume trends to be fixed but rather variable over time.

The Ethics in Research Committee of the Hospital de Clínicas de Porto Alegre (No. 100056) has approved the use of information from surveillance databases for the investigation of chronic diseases by the Collaborative Center for the Surveillance of Diabetes, Cardiovascular and Other Chronic Diseases of the Federal University of Rio Grande do Sul. Given that databases employed had no personal identifiers, no patient consent was necessary.

## Results

A total of 294,203 deaths due to diabetes were officially reported between 1996 and 2011 (Table 1). The type of diabetes was unspecified for the vast majority (91.2 %) of deaths. Acute complications (ICD code final digit .0 or .1) were responsible for 10.6 % of reported deaths, renal complications 19.1 %, peripheral circulatory complications 6.1 %, other complications 12.4 %, while deaths “without complications” corresponded to 51.9 % of the total deaths.

**Table 1:** Number of deaths due to diabetes by specific ICD-10 causes of death

ICD Code number	E10	E11	E12	E13	E14	Total	
	DM1	DM2	MR DM	Other	Unspecified		
ICD Code final digit	N	N	N	N	N	N	%
.0 With coma	1008	730	28	48	11,925	13,739	4.7
.1 With ketoacidosis	1134	503	12	71	15,500	17,220	5.9
.2 With renal complications	1397	3350	35	41	51,261	56,084	19.1
.3 With ophthalmic complications	17	17	2	2	108	146	0.0
.4 With neurological complications	91	79	3	2	630	805	0.3
.5 With peripheral circulatory complications	519	988	10	37	16,368	17,922	6.1
.6 With other specified complications	159	241	524	19	3346	4289	1.5
.7 With multiple complications	1369	944	132	60	8180	10,685	3.6
.8 With unspecified complications	1073	1014	22	150	18,438	20,697	7.0
.9 Without complications	2823	7084	35	88	142,586	152,616	51.9
Total N	9590	14,950	803	518	268,342	294,203	100.0
%	3.3	5.1	0.3	0.2	91.2	100.0	

Mortality Information System (SIM), Brazil, 1996–2011

*DM1* type 1 diabetes, *DM2* type 2 diabetes, *MR DM* malnutrition-related diabetes

We present on Table 2 the proportions of each group of “garbage codes” redistributed to diabetes. Non-specified causes of renal failure were the most likely to be so redistributed: 57.3 % of deaths due to this cause (2979 deaths in 1996 and 2911 in 2011) were redistributed to diabetes.

**Table 2:** GBD-2010 “garbage code” groups [15] having more than 1 % of their codes redistributed to diabetes

Garbage code group^a^	ICD-10 codes^b^	Proportions from each code group redistributed to diabetes
Renal failure	N17–N19	57.3
Disorders of electrolyte and fluid balance	E86–E87	6.43
Osteomyelitis	M86	4.96
Ill-defined infectious diseases	A59–A60.0, A60.9, A63–A64, A71–A74, B07–B09, B35–B36, B74.4–B74.8, B75, B85–B88, B95–B97	4.87
Disseminated intravascular coagulation, cardiac arrest, acute or unspecified respiratory failure	D65, I46, J96.0, J96.9	4.81
Ill-defined diseases of the musculoskeletal system and connective tissue	M10–M11, M15–M25, M40, M45, M47–M48, M50–M60, M65–M67, M70–M71, M75–M79, M95–M99	4.53
Ill-defined diseases of the skin and subcutaneous tissue	L03, L04, L20–L30, L45, L50, L52–L68, L70–L85, L90–L92, L98	4.50
Ill-defined diseases of the nervous system	G43–G44, G47–G52, G54, G56–G58	4.50
Ill-defined diseases of diseases of the genitourinary system	N39.3, N40, N45–N46, N60, N84–N92, N95, N97	4.38
Ill-defined diseases of the digestive system	K00–K11, K14	4.24
Ill-defined diseases of the blood and blood-forming organs and certain disorders involving the immune mechanism	D10–D24, D26–D31, D35–D36	4.11
Ill-defined diseases of the ear and mastoid process	H00–H02, H04–H05, H10–H11, H15–H18, H20–H21, H25–H26, H30–H31, H33–H35, H43–H47, H49–H57, H60–H61, H69, H71–H74, H80–H81, H83–H93	3.69
Ill-defined diseases of the respiratory system	J30, J33, J34.2, J35	3.03
Ill-defined mental and behavioral disorders	F30–F33, F34.1, F40–F48, F51–F53, F60–F99	2.62
Essential and secondary hypertension	I10, I15	1.80
Ill-defined congenital malformations, deformations and chromosomal abnormalities	Q16–Q18, Q36, Q54, Q65, Q67–Q68, Q72–Q74, Q82–Q83	1.15
Encephalopathy and cerebral edema	G92, G93.1–G93.6	1.10

^a^Lozano et al. [15]

^b^Defined by the research group

Table 3 demonstrates the fraction of ill-defined (chapter XVIII-ICD10) codes that were then redistributed to diabetes. In all, 55,195 deaths officially coded as ill-defined were redistributed to diabetes. The percent that was redistributed varied considerably by region and sex, being less in the Center-West region and women having approximately double the percent redistributed of men.

**Table 3:** Percent of R codes* redistributed to diabetes

Year	Region	Redistributed to diabetes	
		Men (%)	Women (%)
1996–2005	North	4.87	8.81
1996–2005	Northeast	5.97	10.54
1996–2005	Southeast	6.61	9.73
1996–2005	South	4.60	8.70
1996–2005	Center-West	1.72	2.38
2006	North	4.57	10.00
2006	Northeast	5.11	7.04
2006	Southeast	7.82	10.83
2006	South	2.79	5.26
2006	Center-West	6.08	8.89
2007	North	3.43	7.59
2007	Northeast	5.95	11.63
2007	Southeast	7.74	11.35
2007	South	3.24	7.75
2007	Center-West	0.29	0.92
2008	North	4.43	4.00
2008	Northeast	6.89	11.44
2008	Southeast	7.23	10.47
2008	South	2.46	7.74
2008	Center-West	1.28	2.78
2009	North	5.33	8.67
2009	Northeast	6.36	12.98
2009	Southeast	7.56	10.12
2009	South	5.81	9.38
2009	Center-West	1.06	0.00
2010	North	5.52	9.63
2010	Northeast	5.32	9.76
2010	Southeast	5.81	9.13
2010	South	6.16	8.83
2010	Center-West	1.76	1.34
2011	North	5.31	11.91
2011	Northeast	6.42	11.10
2011	Southeast	4.89	7.72
2011	South	4.63	9.79
2011	Center-West	3.27	5.63

Brazil, 2006–2011

* ICD-10 Chapter XVIII: Symptoms, signs and abnormal clinical and laboratory findings, not elsewhere classified

As a result of correction for underreporting of deaths, a total of 41,472 deaths were then added, the net addition being most pronounced for the North and the Northeast regions.

Table 4 shows the effect of all these corrections on the total number of deaths and on the age-standardized mortality rates for 1996 and 2011, the first and last years analyzed. In 1996, the corrections resulted in 11,616 additional deaths due to diabetes, an increment of 85 % in total deaths. With improvements in mortality reporting, less deaths were redistributed by 2011, 7334, representing an increment of 30.7 % in total deaths due to diabetes. Correction exerted a greater effect on rates in the North and Northeast, regions with the highest diabetes mortality rates in 2011 for both men and women.

**Table 4:** Effect of correction for underreporting of deaths and ill-defined causes of death on the number of deaths and age-adjusted mortality rates (/100,000)* due to diabetes in adults, by region and sex

Region	1996					2011				
	Before correction		After correction		Change	Before correction		After correction		Change
	n	Rate	n	Rate	%	n	Rate	n	Rate	%
Men										
North	202	14.1	639	44.1	212.8	820	31.5	1248	47.5	50.8
Northeast	1187	18.3	3844	59	222.4	3599	35.3	4953	48.6	37.7
Southeast	3634	30.6	5521	46.2	51.0	5218	28	6870	36.8	31.4
South	954	22.8	1358	32.4	42.1	1787	26.7	2203	33	23.6
Center-West	305	19.2	527	33.1	72.4	744	25.9	975	33.8	30.5
Brazil	6282	24.6	11889	46.2	87.8	12168	29.7	16249	39.6	33.3
Women										
North	205	15.8	661	49.3	212.0	784	31.2	1146	45.2	44.9
Northeast	1600	21.6	5139	69.3	220.8	3787	32.3	4980	42.6	31.9
Southeast	4023	30.1	5449	40.6	34.9	4802	22.3	6010	27.9	25.1
South	1059	23	1444	31.2	35.7	1625	21.7	1949	26.1	20.3
Center-West	319	21.2	522	34.1	60.8	749	24.9	915	30.4	22.1
Brazil	7206	25.5	13215	46.6	82.7	11747	25.4	15000	32.4	27.6

SIM, Brazil, 1996 and 2011

* Mortality rates standardized to the age structure of the population of Brazil in 2010

**Fig. 1:**
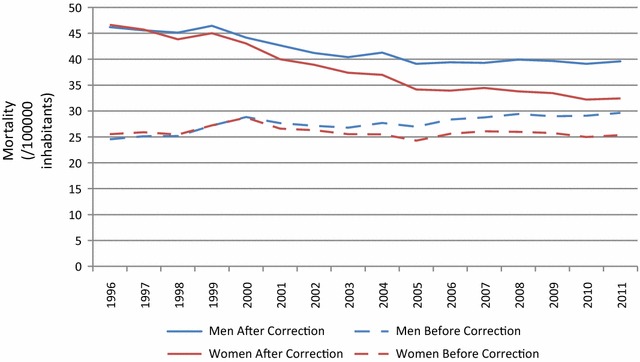
Trends in age-standardized diabetes mortality rates (/100,000) before and after correction for men (*blue*) and women (*red*). SIM, Brazil, 1996–2011. Rates are standardized to the 2010 age distribution of the Brazilian population

Figure 1 shows the trend in mortality due to diabetes for Brazil, from 1996 to 2011. Comparing the uncorrected (dashed lines) with the corrected (solid lines) rates, we observe that a picture of low and if anything rising rates is transformed into one of higher rates in decline, especially for women. The decline is approximately twice as large in women (1.01 deaths/100,000/year, 2.2 %/year, P < 0.001; red line) as men (on average .49 deaths/100,000/year, 1.1 %/year, P < 0.001; blue line), leading to a 30.5 % drop for women, and a 14.3 % drop for men, over the 15 year period.

**Fig. 2:**
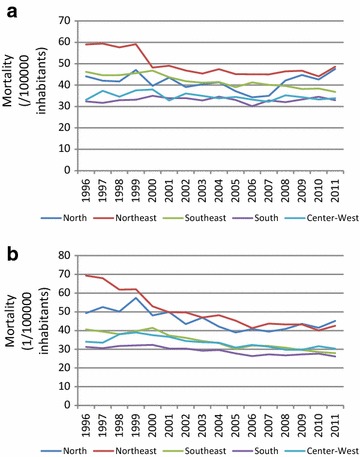
Trends in diabetes mortality in Brazil, by region, 1996–2011. *Panel* **a** men, *Panel* **b** women. Rates are standardized to the 2010 age distribution of the Brazilian population

Figure 2 shows this decline, separately in each of Brazil’s five regions. Declines were largest in both relative and absolute terms in the Northeast and Southeast. In the Northeast, rates declined 1.9 %/year and 38.6 % overall for women and 0.74 %/year and 17.7 % overall for men.

**Fig. 3:**
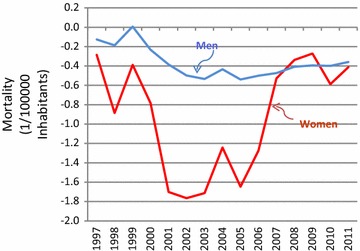
Annual change in diabetes mortality, after correction of the data, according to the state-space model. Brazil, 1996–2011

Figure 3 show the evolution of the trend over time according to the state-space model, which estimates the change in rate in each year compared to the previous year. The trend over time is one of annual declines of varying size since the beginning of the series. In the period from 1998 to 2006, the decrease from 1 year to the next was more accentuated for women. Since 2007, little change in rate has been observed for both men and women.

## Discussion

Our findings demonstrate a decline in standardized diabetes mortality (ICD-10 codes E10-E14) of approximately 1 %/year for men and 2.2 %/year for women from 1996 to 2011 in Brazil. This decrease in mortality due to diabetes became apparent only after corrections for ill-defined causes of death and under registration. The rate of decline first accelerated and then decelerated over the period. The trend was observed in all regions, and attenuated regional inequalities in diabetes mortality in relative and absolute terms.

These findings highlight the need to incorporate the progressive improvements in the mortality system in Brazil over the last two decades when describing trends during the period. At the end of our series, in 2011, correction for remaining mortality system deficiencies resulted in an increment of approximately 30 % to deaths reported as being due to diabetes. In 1996, the increment was approximately 85 %. Without these corrections to account for improvements in the quality of mortality reporting, the decline was hidden by the increasing coverage and the increasingly correct attribution of diabetes as the underlying cause in the mortality registry. The inclusion of these corrections, as seen in Fig. 1, changed the interpretation of trends over the period from one of stability in women and a slight increase in men, to declines in age-adjusted mortality in both sexes.

Earlier studies of diabetes mortality in Brazil, focused on state capitals to minimize the limitations of the mortality information system for Brazil as a whole and covering the initial years of our series, have found varying declines in mortality in some capitals in the Northeast and Southeast [8, 9].

Analyses of mortality trends in Brazil taking into account the variability across space and time of the insufficiencies of the system has received great attention over the past 5 years. Applying corrections for under reporting and ill-defined causes of death to mortality due to the four main non communicable diseases in Brazil revealed a sharp decrease for cardiovascular and chronic respiratory diseases and a modest decrease for diabetes and cancer [12]. Progressive refinements in the methods for these corrections also revealed modest declines in standardized mortality due to diabetes: 0.89 %/year from 2000 to 2009 [22]; 1.7 %/year from 2000 to 2011 [13]; and 1.64 %/year in women and 0.40 %/year in men from 2000 to 2011 [23].

Our findings, more detailed and focused in diabetes, were based on more updated correction algorithms, including redistribution of deaths initially coded in garbage codes. The trend we found, producing a U-shaped curve of rate change, particularly among women, with a deceleration from 2005 onward, not previously reported, deserves reflection.

These rates, and their change over time, summarize the effects of competing forces within a very complex epidemiologic picture. Over this period, the prevalence of diabetes has increased considerably. Data are sparse with respect to the prevalence of diabetes in the 1990s in Brazil, almost always being based on self-report. The self-reported prevalence, estimated from a national survey in 1998 of those 20 or older, was 3.3 % [24]. It increased almost 100 %, to 6.2 % in the Brazilian National Health Survey (Pesquisa Nacional de Saúde, or PNS) of those 18 or older, conducted in 2013 [5]. The increase in prevalence may have resulted from greater diagnosis as well as from greater incidence of diabetes.

Thus, a considerably larger fraction of the population was at risk to die of diabetes and to have this reported as a cause of death in more recent years. As such, improving mortality among those with diabetes competes with the growing prevalence of diabetes to define the mortality trend. Further declines in diabetes mortality may be difficult to achieve if the growing prevalence of diabetes persists. The fact that the decline was greater in women may, in part, reflect that the prevalence of diabetes is not increasing as quickly in women during this period. Comparing results from nationally representative household surveys demonstrate that the annual rate of increase in the prevalence of self-reported diabetes was 9 % in men while only 6.3 % in women from 1998 to 2013 [5, 25].

The regional differences we observed in trends, particularly in the Northeast where absolute declines in women were double the national average and in men approximately 60 % greater, suggest that actions by the SUS, the Brazilian National Health System, to reduce inequities are being effective in terms of the care of those with diabetes. It is worth nothing that Alves et al., who investigated trends by state instead of region, found heterogeneity across states within the same region [23].

Possible reasons for the declines in mortality due to diabetes should be considered. Over this period the SUS expanded its coverage greatly, particularly in terms of primary care. In the 1990´s a National Diabetes Plan worked especially to guarantee greater access to insulin. In 2001, the national Plan to Reorganize Care of Hypertension and Diabetes Mellitus was instituted, focused on redirecting the care of diabetes from the hospital to primary care [26]. The National Program of Pharmaceutical Provision for Hypertension and Diabetes was created in 2002. This Program, along with subsequent laws and regulations, has resulted in a progressively larger distribution of medicines and medical supplies, free of charge, to those with diabetes [27]. Mortality from the acute causes of diabetes has fallen 71 % from the beginning of the 1990s to 2010 [28]. As these acute causes of death are those most sensitive to access and availability of insulin and other medications, they most likely result from the above-mentioned actions as well as the increasing organization of emergency care facilities, transport, and hotline support systems [29]. Undoubtedly, the increased standard of living, the rise of the Brazilian middle class, decreasing poverty and efforts to eradicate severe poverty such as the cash transfer program *bolsa família* may have also played a difficult-to-estimate but important role in the decline [30]. Unfortunately, given that diabetes type was “unspecified” for 91 % of deaths, the data do not permit the description of declines for specific types of diabetes.

Additionally, and also in part due to the above mentioned reasons, mortality from chronic diseases in general and cardiovascular diseases in particular, the major causes of diabetes deaths, has fallen considerably [12]. Declining rates of smoking, a major risk factor for complications of diabetes, also fell considerably over this period. Improved care of diabetes has been postulated to explain documented increased survival among those with diabetes in various high income countries, including Sweden, the UK and Taiwan [3]. In the US, findings from the National Health Interview Survey (1997–1998, 1999–2000, 2001–2002, and 2003–2004 for adults aged 18 years and older show that among diabetic adults, the CVD death rate declined by 40 % (95 % CI 23–54) and all-cause mortality declined by 23 % (10–35) between the earliest and latest samples. The excess CVD mortality rate associated with diabetes (i.e., excessive when compared with rates of nondiabetic adults) decreased by 60 % (from 5.8 to 2.3 CVD deaths per 1000) while the excess all-cause mortality rate declined by 44 % (from 10.8 to 6.1 deaths per 1000) [31].

Thus, our findings of decreasing mortality due to diabetes are likely to result from improved care to diabetes in Brazil over the last two decades. Yet, the deceleration observed more recently may indicate that further declines will only occur with further strengthening in the organization of care of those with diabetes. Moreover, primary prevention efforts, including population-oriented public health actions such as food and agricultural policies aimed at making healthy choices easier, are much needed to stem the increase in diabetes incidence.

Strengths and limitations of our investigation merit comment. Among the strengths is the use of methodologies to correct for deficiencies in the Brazilian system of death registry which are more in consonance with the GBD project, most specifically the incorporation of a recent GBD approach to garbage code distribution. The GBD also is evolving in its methodologies, and future changes in the redistribution of garbage codes are anticipated.

A limitation of this study is the fact that deaths coded in E10-E14, when considered within the broader framework of the multicausality of disease, are only part of the overall mortality that can be logically attributed to diabetes. Many deaths from complications which the epidemiologic literature suggests can be attributed to diabetes—heart disease, stroke, renal failure, and even cancer [32] and some infectious diseases such as tuberculosis—[33] will never formally be indicated in mortality registry systems as due to diabetes. Studies suggest that mortality attributable to diabetes could be from 50 % to as much as three times greater than that calculated using diabetes as the underlying cause of death [34–38]. Since we have only analyzed the underlying cause of death, without considering the remaining causes listed in part I of the death certificate, frequent direct causes such as myocardial infarction, stroke or pneumonia were not included in this report. Further, due to the process of coding, even if such direct causes of deaths were present on the death certificate, the diabetes ICD code chosen would most likely end in .9 (“without complications”), as no specific additional digit is available for the coding many of the important specific complications. Nevertheless, we believe that the ICD, inadequate as it is, still provides the basic information necessary to describe trends in diabetes mortality, the objective of our manuscript.

Another limitation is related to the methods used to assess completeness of death registration using indirect demographic methods with some controversial assumptions like absence of migration, and constancy of incompleteness across all ages. These assumptions could potentially affect measures of completeness and the estimates of mortality rates [39].

In conclusion, our data suggest that standardized premature mortality due to diabetes, based on death certificate coding, has declined in Brazil over the 15 years from 1996 to 2011. The rate appears to have stabilized in the later years of this series, suggesting that the effect of the increasing prevalence of diabetes now threatens to reverse this trend by outweighing the gains made through better patient care. These data suggest that for the decline to continue, solutions must be found not only to improve diabetes care but also to prevent the current epidemic increase in the incidence of diabetes.
